# Medication-Related Osteonecrosis of the Jaw: A Case Report of an Unusual Side Effect of Adalimumab

**DOI:** 10.1155/2023/5544285

**Published:** 2023-12-15

**Authors:** Laura Sisalli, Francesco Giordano, Andrea Chiacchio, Alfonso Acerra, Mario Caggiano

**Affiliations:** Department of Medicine Surgery and Dentistry, “Scuola Medica Salernitana”, Via Allende, Baronissi, Italy

## Abstract

**Background:**

Medication-related osteonecrosis of the jaw (MRONJ) is a serious adverse drug reaction characterized by progressive bone destruction and necrosis of mandibular and/or maxillary jaw bone that occurs in patients undergoing treatments with antiresorptive drugs such as bisphosphonates or denosumab, antiangiogenic agents such as bevacizumab, or other kinds of monoclonal antibodies such as rituximab and ipilimumab, for different oncologic and nononcologic diseases. The aim of this study was to report a case of MRONJ in a patient affected by rheumatoid arthritis disease in treatment with adalimumab. *Case Presentation*. A 70-year-old female patient affected by rheumatoid arthritis (RA), who had been undergoing adalimumab (40 mg subcutaneous injection) every two weeks for 5 years, with no history of antiresorptive or antiangiogenic agent administration, came to our attention for intraoral necrotic bone exposures of the anterior mandible. After drug withdrawal and antibiotic cycles, the patient underwent surgical treatment with bone resection and debridement of necrotic tissues. After an observation period of 8 months, a complete healing without signs of recurrence was detected.

**Conclusions:**

Based on this study, a correlation between adalimumab and MRONJ is possible. Therefore, we believe that an oral cavity examination should be done in every patients, before starting therapy with adalimumab, to possibly avoid MRONJ onset. Further studies are required to confirm the role of adalimumab in MRONJ.

## 1. Background

Medication-related osteonecrosis of the jaw (MRONJ) is a serious adverse reaction experienced by some individuals to some drugs commonly used in the treatment of tumor pathologies and osteoporosis (e.g., bisphosphonates, denosumab, and antiangiogenic agents) and involves the progressive destruction of bone in the jaw or maxilla [1, 2]. It is characterized by progressive bone destruction and necrosis of mandibular and/or maxillary in subjects exposed to the treatment with drugs that are known to have an increased risk of disease, in the absence of a previous radiant treatment [3]. Bisphosphonates (BP) and denosumab are the most involved medication in MRONJ onset because of the impairment they cause to bone metabolism [4–9].

In the last few years, besides the antiresorptive drugs, several other categories of drugs have been reported as possible inducing agents of osteonecrosis of the jaw (ONJ) (e.g., tyrosine kinase inhibitors (TKIs), mechanistic target of rapamycin (mTOR) inhibitors, and immunomodulatory drugs). Hence, the clinician's role in pharmacovigilance should be considered more relevant than ever [10–13].

Monoclonal antibodies are not only used for oncologic disease but also for other systemic diseases such as rheumatoid arthritis (RA) that is a chronic, ankylosing, and progressive inflammatory polyarthritis, with autoimmune pathogenesis and unknown etiology, mainly affecting synovial joints [14]. The global prevalence of RA was 460 per 100,000 population with a 95% prediction interval (0.06–1.27%), with variations due to geographical location and study methodology [15]. RA typically occurs at age of 35–50 years, and females are affected 2.5 times more frequently than males [16].

It has been found that tumor necrosis factor-*α* (TNF-*α*) has a critical role in the pathogenesis of RA, and its inhibition is an effective treatment [17–19].

Adalimumab is a monoclonal antibody that binds specifically to TNF-alpha and blocks its interaction with the p55 and p75 cell surface TNF receptors. Within inflammatory and immune response, one of the cytokines involved is TNF itself. Since its initial approval by the Food and Drug Administration (FDA) for treatment of rheumatoid arthritis, adalimumab has been authorized for several other diseases, including hidradenitis suppurativa, iritis, ankylosing, psoriatic, and juvenile idiopathic arthritis; plaque psoriasis; ulcerative colitis; and Crohn's disease [20–22].

Adalimumab was usually used in the combination with methotrexate (MTX). But it can also be used alone when treatment with MTX is contraindicated [20]. To date, only one case of osteonecrosis of the jaw in a RA patient following a course of adalimumab therapy has been reported [21].

Generally, MRONJ develops as a result of local infection or trauma to bone or soft tissue and rarely spontaneous occurrence [2]. Some studies have shown that localized periodontal or dental disease may precede the onset of MRONJ [23]. Dentists, as part of a multidisciplinary team, have a critical role in preventing MRONJ because the development of this condition can be reduced through prophylactic dental care and the maintenance of good oral hygiene [24–27]. The importance of prevention is also critical in patients who have to take adalimumab, as this study poses the need to further investigate the association between it and the adverse reaction of MRONJ.

The aim of this study was to report a case of MRONJ in a female patient affected by rheumatoid arthritis disease who had been undergoing adalimumab and methotrexate administration for several years.

## 2. Case Presentation

On March 2022, a 70-year-old female patient was referred to the Dental Unit of the San Giovanni di Dio and Ruggi d'Aragona University Hospital of Salerno for intraoral necrotic bone exposures of the anterior mandible, associated with submandibular swelling, pus discharge, and pain underneath the lower right and left central incisors since 6 months prior to the visit.

The patient's medical history included smoking habit (10 cigarettes per day for 46 years); thrombophlebitis of the right lower limb treated with rivaroxaban (20 mg orally, once a day); chronic autoimmune rheumatoid arthritis treated with intramuscular injection of methotrexate (10 mg, once a week) that was started since March 2009; subcutaneous administration of adalimumab (40 mg, once every 2 weeks) since January 2017; folic acid (5 mg orally, once a day) for her iron deficiency anemia; and atorvastatin (40 mg orally, once a day) for her hypercholesterolemia (Table 1).

**Figure 1:**
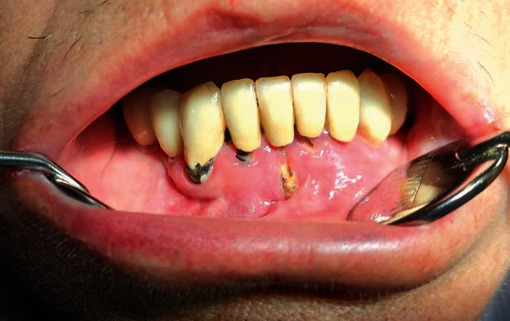
Intraoral necrotic bone exposures of the anterior mandible.

Clinical examination of the oral cavity revealed the presence of 3 implant-supported prosthesis extended from 16 to 13, 34 to 36, and 44 to 45 and 2 fixed tooth-supported prosthesis extended from 12 to 23 and from 32 to 43. A 1.5 cm bone exposure area with pus discharge was noticed in the parasymphyseal zone, from 31 to 41 (Figure 1). An atrophic area on the dorsum of the tongue was also detected.

**Figure 2:**
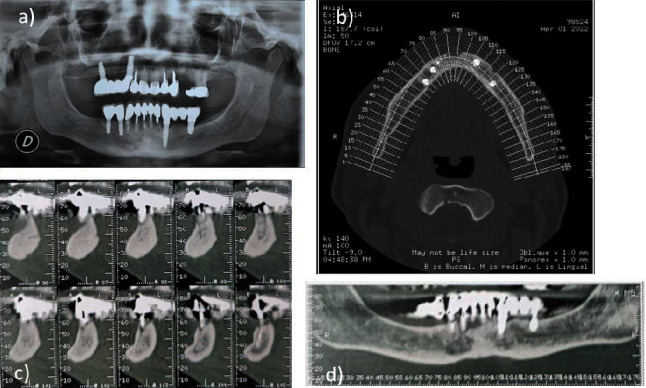
Radiographic examinations performed in March 2022: (a) the OPT showed the presence of several dental treatments and an area of radiolucency below the lower incisors; CBCT revealed the presence of bone sequestration area; (b) axial cuts of the CBCT scan; (c) sagittal cuts of the CBCT; (d) coronal cuts of the CBCT.

Orthopantomography (OPT) and cone-beam computed tomography (CBCT) showed the presence of bone sequestration area extended from the lower left lateral incisor (32) to the lower right lateral incisor (42) and a periapical radiolucency around the apex of the lower central incisor (31 and 41), site of an underfilled root canal therapy (Figure 2).

Based on these clinical and radiological findings, a provisional diagnosis of MRONJ was formulated, and the lesion was classified as stage 2 according to the American Association of Oral and Maxillofacial Surgeons staging system [28] (Table 2).

Immediately, adalimumab treatment was suspended after consulting her rheumatologist, and the patient started an antibiotic therapy course with amoxicillin and clavulanic acid (1 g orally, three times a day) assumed for 6 days. Patient was advised to use chlorhexidine digluconate 0.20% mouthwash two times a day for 10 days. On the follow-up, scheduled at the end of the prescribed drug therapy, the patient showed an evidence of clinical improvement, i.e., the swelling and pus discharge had resolved, and the MRONJ reversed from stage 2 to stage 1. The local therapy with chlorhexidine digluconate 0.20% mouthwash (twice a day) and ozonized mouthwash (twice a day) was prescribed as maintenance drug therapy for another 2 months [29]. The patient was reviewed every 3 weeks.

During an examination in June 2022, the mobilization of the exposed bone and the stabilization of MRONJ stage 1 were detected; considering this novel finding, the resection of the area of bone sequestration and extraction of the mandibular anterior teeth (32, 31, 41, 42, and 43) were performed under local anesthetic infiltration and antibiotic therapy (1 g orally, two times a day) assumed for 6 days from three days before surgical treatment. Furthermore, autologous platelet concentrates as hemostatic agents can improve healing in this case [30]. The surgical specimen was fixed in neutral-buffered formalin, and its histopathological analysis showed areas of bone necrosis and fibrinoleukocyte tissue. Following the surgical treatment, local therapy with chlorhexidine digluconate 0.20% mouthwash (twice a day) for 2 weeks was prescribed and preventive oral hygiene care was given to the patient. The patient enters in a follow-up program every 3 months.

**Figure 3:**
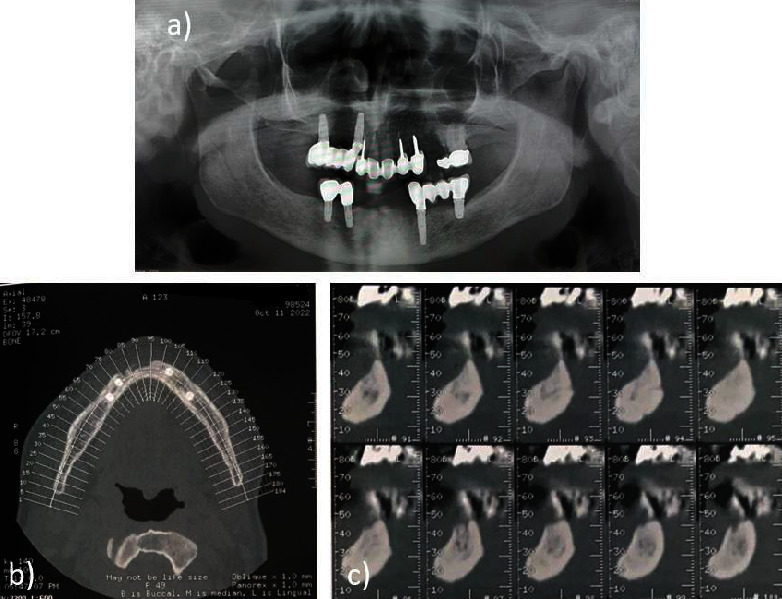
Radiographic examinations performed in October 2022: (a) the OPT showed an absence of radiolucency in the region of the lower incisors; the CBCT showed a complete healing of the bone tissue; (b) axial cuts of CBCT; (c) sagittal cuts of CBCT.

**Figure 4:**
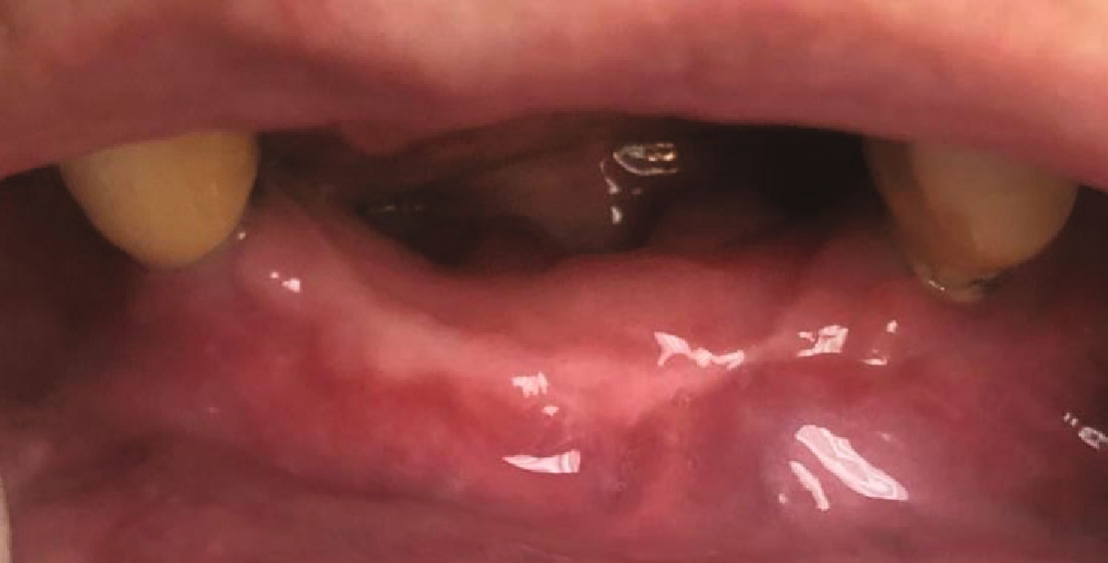
Soft tissue healing 9 months after surgical treatment.

**Figure 5:**
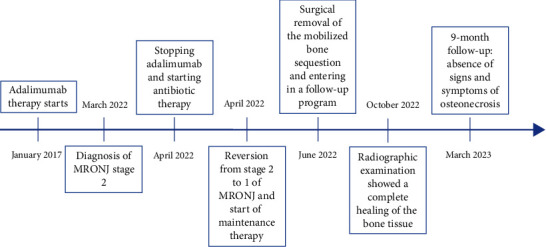
Patient's medical history timeline.

On October 2022, the patient showed intraoral wound healed without complications and without recurrence confirmed by radiographic control examination (Figure 3). Moreover, at the last follow-up examination in March 2023, the absence of clinical signs of ONJ and complete healing of the oral soft tissues were confirmed (Figures 4 and 5).

After complete healing, the edentulous space will be replaced by placement of 2 implants in area 3.2 and 4.3 supporting a 5-unit splinted dental fixed dental prosthesis [31].

## 3. Discussion

The present case report describes MRONJ development in a 70-year-old female affected by RA who was treated for various years with methotrexate and adalimumab. She has never had antiresorptive/antiangiogenic treatments. Adalimumab is a recombinant IgG, anti-TNF-alpha monoclonal antibody, that inhibits the linkage of TNF-alpha (both soluble and membrane-bound) to p55 (TNFR1) and p75 (TNFR2) cell surface receptors, which in turn interferes with cytokine-driven inflammatory processes. TNF mediates activation of nuclear factor kappa-light-chain-enhancer of activated B cell ligand (RANKL) receptors on stromal or osteoblast cells that leads to destruction of cartilage and bone. TNF also plays a role in osteoclast development and activation, which remains in check with TNF blockade. Anti-TNF agents downregulate serum matrix metalloproteinases 1 and 3 (MMP-1 and MMP-3). These factors are important for the effectiveness of TNF inhibitors in the treatment of arthritis [22].

The patient has been treated with methotrexate that is an antifolate metabolite that inhibits DNA synthesis, repair, and cellular replication. At chemotherapeutic dosage, it has been observed that MTX inhibits bone formation and mineralization. It could be related with increased bone resorption, as indicated by increased osteoclast density [32–36].

The patient developed MRONJ that is a critical adverse drug-related reaction characterized by progressive bone destruction and mandibular and/or maxillary necrosis in patients treated with drugs that are known to have an increased risk of disease, in the absence of a previous radiant treatment [2].

Studies showed that bisphosphonates and denosumab have been most associated with this disease, although antiangiogenic agents have also been reported to cause MRONJ [1, 2]. More recently, a number of reports have described MRONJ development in patients treated with tumor necrosis factor alpha (TNF-alpha) inhibitors such as infliximab, adalimumab, golimumab, and etanercept [37, 38].

Exposed necrotic bone in the oral cavity is the most common clinical sign, with a history of pain (odontalgia, weight-bearing bone pain, myogenic pain, sinus pain, and trigeminal-type pain), and interruption of local wound healing processes [39]. Consequently, our patient reported pain in the lower middle dental arch for unknown period and had exposed bone in this area. Other signs typically but less frequently described are intra- and extraoral soft tissue swelling, intra- and extraoral fistula formations, nasal discharge, sinusitis ipsilateral to necrosis, purulent discharge, odontogenic abscesses, and halitosis [39]. In our case, the signs and symptoms presented were swelling of the middle lower gingiva, abscess collection, and sinusitis of right maxillary sinus. In the most severe cases, an extension of the necrotic process to the basal bone, pathological fractures, or nerve impairment, particularly at the mandibula may occur [39], but it was not found in our case. Generally, the anatomic risk factors of the mandible have as a result that the most affected part is the jaw in 75% of the cases, and in the patient case, the lower jaw was involved, unilaterally [2].

In this case report, the patient initially had an intraoral necrotic bone exposures of the anterior mandible, accompanied with pus discharge with an extension to the mandibular incisors that were affected by endodontic failure (underfilled root canal therapies) and surrounded with areas of periapical radiolucency observable on CTCB [40]. A review published by Daokar in 2013, reported that the development of radiographically detectable periapical radiolucency combined with incongruous filling of the root canal, is a sufficient element to consider the endodontic therapy failed [41]. Bacterial residuals can induce inflammatory phenomena that could be considered as the trigger of the ONJ development in our case report. Several studies had proven the presence of actinomycetes within biopsies of necrotic bone resected from patients with MRONJ. It was interesting to note that, in our case report, the sequestered bone was strictly related to areas of periapical radiolucency around the lower central incisors [42, 43].

Although surgical therapy with extraction of the lower incisors and removal of the necrotic bone was immediately indicated [28], we preferred to administer drug therapy to reduce bacterial contamination and improve the healing of surrounding soft tissue [44]. The approach applied in the current study followed the guidelines on the MRONJ management described by the American Association of Oral and Maxillofacial Surgeons, and it has been recommended at any stage of the MRONJ [45, 46]. At the end of drug therapy, MRONJ had regressed from stage 2 to stage 1, with an improvement of gingival soft tissue health and the absence of suppuration. The surgery was performed under more predictable conditions that guaranteed a first intention healing to avoid postoperative infection and wound dehiscence caused by the presence of bacteria into the surgical site [47–49].

There are no studies in the literature that show a relation between the only administration of methotrexate and the MRONJ [50]. Only one case report described a possible correlation between adalimumab and MRONJ [21]. Specifically, Cassoni et al. described a case of 63-year-old female with diagnosis of MRONJ stage 0 following a course of adalimumab therapy. Preidl et al. described a case of MRONJ associated with adalimumab and bisphosphonate. Consequently, this medication could promote manifestation of osteonecrosis [51].

In conclusion, although most MRONJ are related to the administration of bisphosphonates, as well as denosumab, clinicians need to be aware that the monoclonal antibody might also cause complication. The intent of this case report is to share the possible cause-effect relationship of osteonecrosis of the jaw in a seventy-year-old patient who was treated both with adalimumab and methotrexate. Further studies are needed to investigate the relationship between the side effects of adalimumab and MRONJ.

## Figures and Tables

**Table 1:** Drugs consumed by the patient.

Drugs	Therapy	Treatment indication
Adalimumab	40 mg, once every two weeks, taken for 6 years	Rheumatoid arthritis
Methotrexate	10 mg, once a week, intramuscular injection, taken for 14 years	Rheumatoid arthritis
Rivaroxaban	20 mg, once a day	Thrombophlebitis
Folic acid	5 mg, once a day	Iron deficiency anemia
Atorvastatin	40 mg, once a day	Hypercholesterolemia

**Table 2:** Staging of medication-related osteonecrosis of the jaw according to the American Association of Oral and Maxillofacial Surgeons. Reproduced from Ruggiero et al. [28].

At risk	No apparent necrotic bone in patients who have been treated with oral or intravenous bisphosphonates
Stage 0	No clinical evidence of necrotic bone but nonspecific clinical findings, radiographic changes, and symptoms
Stage 1	Exposed and necrotic bone or fistula that probes to bone in patients who are asymptomatic and have no evidence of infection
Stage 2	Exposed and necrotic bone or fistula that probes to bone associated with infection as evidenced by pain and erythema in the region of exposed bone with or without purulent drainage
Stage 3	Exposed and necrotic bone or a fistula that probes to bone in patients with pain, infection, and ≥1 of the following: exposed and necrotic bone extending beyond the region of alveolar bone (i.e., inferior border and ramus in mandible, maxillary sinus, and zygoma in maxilla) resulting in pathologic fracture, extraoral fistula, oral antral or oral nasal communication, or osteolysis extending to inferior border of the mandible or sinus floor

## Data Availability

The data used to support the findings of this study are available from the corresponding author upon request.
